# The Effect of Cardiovascular Comorbidities on Women Compared to Men: Longitudinal Retrospective Analysis

**DOI:** 10.2196/28015

**Published:** 2021-10-04

**Authors:** Elma Dervic, Carola Deischinger, Nils Haug, Michael Leutner, Alexandra Kautzky-Willer, Peter Klimek

**Affiliations:** 1 Section for Science of Complex Systems Center for Medical Statistics, Informatics and Intelligent Systems Medical University of Vienna Vienna Austria; 2 Complexity Science Hub Vienna Vienna Austria; 3 Department of Internal Medicine III Clinical Division of Endocrinology and Metabolism Gender Medicine Unit, Medical University of Vienna Vienna Austria; 4 Gender Institute Gars am Kamp Austria

**Keywords:** gender gap, sex differences, cardiovascular diseases, acute myocardial infarction, chronic ischemic heart disease, gender, diabetes, smoking, risk factors, comorbidities

## Abstract

**Background:**

Although men are more prone to developing cardiovascular disease (CVD) than women, risk factors for CVD, such as nicotine abuse and diabetes mellitus, have been shown to be more detrimental in women than in men.

**Objective:**

We developed a method to systematically investigate population-wide electronic health records for all possible associations between risk factors for CVD and other diagnoses. The developed structured approach allows an exploratory and comprehensive screening of all possible comorbidities of CVD, which are more connected to CVD in either men or women.

**Methods:**

Based on a population-wide medical claims dataset comprising 44 million records of inpatient stays in Austria from 2003 to 2014, we determined comorbidities of acute myocardial infarction (AMI; International Classification of Diseases, Tenth Revision [ICD-10] code I21) and chronic ischemic heart disease (CHD; ICD-10 code I25) with a significantly different prevalence in men and women. We introduced a measure of sex difference as a measure of differences in logarithmic odds ratios (ORs) between male and female patients in units of pooled standard errors.

**Results:**

Except for lipid metabolism disorders (OR for females [ORf]=6.68, 95% confidence interval [CI]=6.57-6.79, OR for males [ORm]=8.31, 95% CI=8.21-8.41), all identified comorbidities were more likely to be associated with AMI and CHD in females than in males: nicotine dependence (ORf=6.16, 95% CI=5.96-6.36, ORm=4.43, 95% CI=4.35-4.5), diabetes mellitus (ORf=3.52, 95% CI=3.45-3.59, ORm=3.13, 95% CI=3.07-3.19), obesity (ORf=3.64, 95% CI=3.56-3.72, ORm=3.33, 95% CI=3.27-3.39), renal disorders (ORf=4.27, 95% CI=4.11-4.44, ORm=3.74, 95% CI=3.67-3.81), asthma (ORf=2.09, 95% CI=1.96-2.23, ORm=1.59, 95% CI=1.5-1.68), and COPD (ORf=2.09, 95% CI 1.96-2.23, ORm=1.59, 95% CI 1.5-1.68). Similar results could be observed for AMI.

**Conclusions:**

Although AMI and CHD are more prevalent in men, women appear to be more affected by certain comorbidities of AMI and CHD in their risk for developing CVD.

## Introduction

Despite the overall higher prevalence of cardiovascular disease (CVD) in men, the gender gap in CVD narrows with age, especially postmenopause [1]. Potential explanations are plentiful and range from a menopausal drop in protective estrogen to certain comorbidities affecting women in a more impactful way [2-5]. However, whether these findings just represent anecdotal evidence or whether they hint at a systematic development in which, given certain risk factors, women are getting an increasingly higher risk for CVD than men is currently unclear. To clarify the role of comorbidities in the CVD gender gap, we aimed at developing a structured approach to screening and identifying sex-specific differences in comorbidities associated with CVD in this analysis.

Some risk factors for CVD are associated with excess risk in 1 sex but not the other. A series of meta-analyses identified smoking [3] and diabetes [2,4] to have a stronger relative effect on CVD risk in women than in men, which, in the case of diabetes mellitus, has been extensively studied. Diabetes mellitus not only doubles the CVD risk but increases the risk by 44% more in females compared to males [6,7]. In the case of chronic kidney disease (CKD) and CVD, dialysis patients have a 50-fold increased CVD mortality rate in comparison to the general population; females, specifically, lose their survival advantage [8]. The female sex has independently been associated with CKD among ST-elevation myocardial infarction (STEMI) patients, which then resulted in a 2-fold relative increase of in-hospital mortality for women in the same study [9]. Sex differences (SDs) in CVD risk amongst patients with respiratory diseases have not been sufficiently investigated so far. In respiratory diseases, both sexes with active asthma were at a 29% higher risk of suffering from an acute myocardial infarction (AMI) compared with adults without asthma in a 2019 study [10]. Although chronic obstructive pulmonary disease (COPD) is more prevalent in men, prevalence and mortality among women have been rising and there are indications that COPD and risk factors for COPD are more detrimental in women than in men. In a study on women and COPD, women were significantly younger and had smoked less than men [11]. Furthermore, although the prevalence of CVD in COPD is higher in men, the impact of CVD on mortality in women with COPD increased in the Obstructive Lung Disease in Northern Sweden (OLIN) COPD study [12].

A more comprehensive quantification of SDs in AMI or chronic ischemic heart disease (CHD) risk in association with other comorbidities, such as respiratory and renal diseases, is still needed. This analysis aimed to fill this knowledge gap by identifying potential gender gaps in comorbidities associated with AMI or CHD and by determining the extent of age-/menopause-related differences in the gender gaps.

## Methods

Both the terms “woman/man” and “female/male” are used in this paper as we investigate SDs. However, through our study design, we cannot rule out an influence of gender aspects (in addition to sex-specific aspects) on disease risk. For the purpose of this study and in line with the previous literature [5,13], “gender gap” is used to describe sex and gender differences in disease risk between men and women.

### Data

Medical claims data of the entire Austrian population were examined with a structured approach to analyze comorbidity networks for female and male patients. This database contains approximately 44 million records, containing for each in-hospital stay in Austria from 1997 until 2014 the patient’s ID, age, date of admission, date of discharge, primary diagnosis, secondary diagnoses, and type of release. The age of patients is given at a resolution of 5 years. The reason for the hospital admission is given by the primary diagnosis. Conditions that coexist at the time of admission are secondary diagnoses. In this study, we considered primary and secondary diagnoses as equally relevant. All diagnoses are recorded in the form of level 3 International Classification of Diseases, Tenth Revision (ICD-10) codes, a medical classification system by the World Health Organization (WHO). This study concentrated on 1080 different ICD-10 codes ranging from A00 to N99. We only extracted the subset of patients who did not have any hospital stays during the 6 years from 1997 to 2002.

The total number of patients in the database is 8,996,916. After extracting the described subset of patients, the total number of patients for this analysis was 3,758,634 (51% women, 49% men). To compare changes that might occur before and after (peri-)menopause, we conducted additional analyses comparing patient groups with ages being above or below the cutoff age of 50 years. The total number of all diagnoses recorded in the selected dataset was 36,358,201 (50.14% diagnoses of female patients, 49.86% diagnoses of male patients). The 5 most frequent diagnoses were hypertension (I10), CHD (I25), type 2 diabetes mellitus (E11), atrial fibrillation and flutter (I48), lipid metabolism disorder (E78) (female: I10, malignant neoplasm of breast [C50], other disorders of urinary system [N39], I25, E11; male: I10, I25, E11, E78, COPD [J44]).

### Co-occurrence Analysis/Relative Risks for Comorbidities

Comorbidities indicate the presence of more than 1 disease in the same person. In our analysis, we investigated all statistically significant co-occurring diseases.

Stratified analysis was performed to adjust for confounding variables (age, time period). The analyzed dataset was stratified by age (10-year age groups) and 6 time windows of 2 years each from 2003 to 2014 (2003-2004, 2005-2006, and so on), resulting in 48 strata for women and men. For each pair of diagnoses for each stratum, a contingency table was built. Contingency tables that contained a sufficient number of patients in each subgroup (>4) were used for computing relative risks (RRs) and the *P*-value for rejecting the null hypothesis that the co-occurrence of 2 analyzed diagnoses is statistically independent.

By using the Cochran–Mantel–Haenszel method [14], we calculated a weighted average of the estimates of the risk ratios and odds ratios (ORs) across the stratified data. To identify sex-specific differences in comorbidities, we identified all comorbidities with an RR higher than 1.5 and a *P*-value smaller than 0.01. Comorbidities with less than 1000 occurrences in female or male patients were excluded.

### Sex Differences

As a test statistic for SDs, we measured the differences of logarithmic ORs between male and female patients in units of pooled standard errors:

SD = [log(ORm) – log(ORf)]/√(SEm^2^ + SEf^2^)

where ORm is the OR for males, ORf is the OR for females, SEm is the standard error of ORm, and SEf is the standard error of ORf.

To test for significant SDs, we tested the null hypothesis that an SD is measured from a normal distribution with zero mean to obtain the SD *P*-value *P*_SD.

We defined 5 significance levels of SDs:

- Not significant
  (SD<=2<==>*P*_SD>=.045)
- Weak
  (2<SD<=3<==>.003<=*P*_SD<.045)
- Substantial
  (3<SD<=4<==>.00006<=*P*_SD<.003)
- Strong
  (4<SD<=5<==>.0001<=*P*_SD<.00006)
- Very strong
  (5<SD<==>*P*_SD<.00001)

### Time Directionality

We calculated the time difference for each pair of diagnoses (A and B) for every patient in the period 2003-2014. The time difference was defined as the difference between the time of the first diagnosis of A and the time of the first diagnosis of B. Patients were separated into 4 groups based on the time interval for each pair: (1) A and B were diagnosed during the same hospital stay and the time difference between A and B was (2) less than 3 months, (3) greater than 3 months and less than approximately 1 year (360 days), or, finally, (4) greater than approximately 1 year.

In each group and for each pair, we counted the number of patients who were first diagnosed with disease A and then disease B, *N*(A→B), and vice versa, *N*(B→A).

For all 4 above-defined time intervals, we calculated the ratio between the number of patients with the direction of “first A then B” relative to “first B then A” and the time order ratio TOR(A→B)=*N*(A→B)/*N*(B→A) to identify the “direction” of each pair.

A TOR(A→B) of <1 (>1) indicates that B (A) tends to occur before A (B). To see whether TOR(A→B) is significantly different from 1, we tested the null hypothesis that *N*(A→B)=*N*(B→A), assuming that both counts stem from a binomial distribution with equal success probability.

## Results

### Baseline Characteristics

We focused on the age group of 20-79 years, with a total of 2,716,967 patients (50.12% women, 49.88% men). As shown in Table 1, 2.02% of all patients were diagnosed with AMI (1.42% women, 2.64% men) and 6.22% of all patients were diagnosed with CHD (4.9% women, 7.58% men).

**Table 1:** Baseline characteristics and prevalence (%) among all patients aged 20-79 years in Austria from 2003 to 2014.

Parameters and diagnoses	All	Female patients	Male patients
All patients	2,716,967	1,361,704	1,355,263
Age (years, mean±SE^a^)	48.53±15.99	47.99±16.2	49.07±15.77
Number of hospital stays (mean±SE)	3.04±4.92	3±4.82	3.08±5.01
Hospital days (mean±SE)	17.52±45.83	16.65±44.38	18.38±47.3
Number of hospital diagnoses (mean±SE)	4.28±4.83	4.12±4.66	4.44±4.99
Obesity and overweight (%)	4.37	4.47	4.26
Disorders of lipoprotein metabolism and other lipidemias (%)	8.46	7.33	9.62
Nicotine dependence (%)	3.16	2.01	4.33
AMI^b^ (%)	2.02	1.42	2.64
CHD^c^ (%)	6.22	4.9	7.58
Asthma (%)	1.3	1.28	1.31
COPD^d^ (%)	3.41	2.69	4.15
Respiratory failure (%)	0.91	0.76	1.06
Diabetes mellitus (%)	6.49	5.93	7.07
Acute kidney failure and CKD^e^ (%)	3.77	3.66	3.88

^a^SE: standard error.

^b^AMI: acute myocardial infarction.

^c^CHD: chronic ischemic heart disease.

^d^COPD: chronic obstructive pulmonary disease.

^e^CKD: chronic kidney disease.

### Overweight and Obesity

In the analysis at hand, the diagnosis of overweight and obesity was more prominently associated with AMI (ORf=3.36, 95% confidence interval [CI]=3.22-3.51 vs ORm=2.8, 95% CI=2.72-2.88, *P*_SD<.0001) and CHD (ORf=3.64, 95% CI=3.56-3.72, ORm=3.33, 95% CI=3.27-3.39, *P*_SD<.0001) in females than in males. To account for potential differences before and after an age considered likely (peri-)menopausal, we investigated the association of overweight and obesity with AMI and CHD in the age group under 50 and over 50 years. The results were similar in both groups; however, the effect of sex on the association between overweight/obesity and AMI and CHD risk was more prominent in the age group of 50 years and above.

### Diabetes Mellitus

Females showed a stronger association of diabetes mellitus with AMI and CHD compared to males (AMI: ORf=2.94, 95% CI=2.77-3.12 vs ORm=2.17, 95% CI=2.09-2.26, *P*_SD<.0001; CHD: ORf=3.52, 95% CI=3.45-3.59, 0.01, ORm=3.13, 95% CI=3.07-3.19, *P*_SD<.0001). The effect was greater in the age group of 50-79 years than in younger patients.

**Figure 1:**
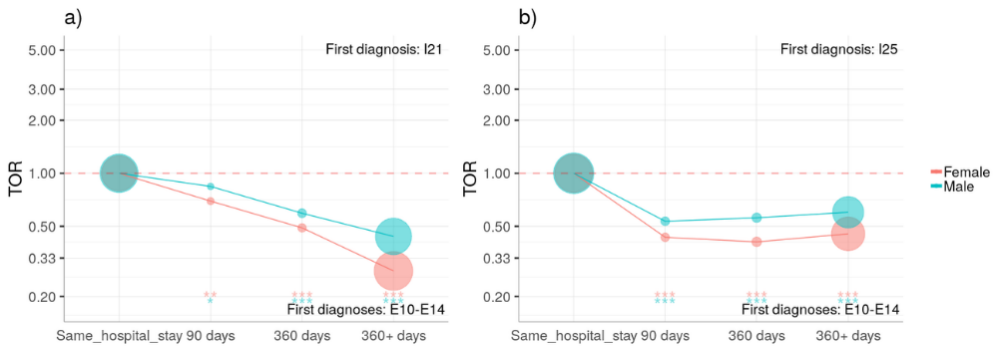
Diabetes mellitus is typically diagnosed before AMI and CHD. We show the time directionality (see Methods) for patients with a diagnosis of diabetes (E10-E14) and (a) AMI (I21) and (b) CHD (I25). The larger the time difference between these two diagnoses, the stronger the dominance of patients first having a diabetes mellitus diagnosis (TOR<1). Significance levels of the TOR are indicated by asterisks (*P<.05, **P<.01, ***P<.0001). AMI: acute myocardial infarction; CHD: chronic ischemic heart disease; TOR: time order ratio.

There is a tendency that diabetes mellitus is diagnosed before AMI and CHD; see Figure 1, where we show the corresponding TORs as a function of time.

The increased risk for female patients with diabetes mellitus to develop AMI or CHD is a well-researched finding. Diabetes mellitus not only doubles the CVD risk but rather adds an additional 44% risk to females compared to males [6,7].

### Acute and Chronic Kidney Disease

**Figure 2:**
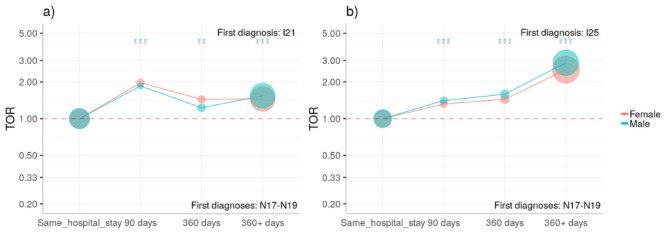
Time directionality analysis for CKD. There is a tendency that patients are first diagnosed with (a) AMI and (b) CHD and then with CKD. Results are shown as in Figure 1 for diabetes. AMI: acute myocardial infarction; CHD: chronic ischemic heart disease; CKD: chronic kidney disease; TOR: time order ratio.

Female patients with acute kidney disease and CKD were more likely to be diagnosed with AMI and CHD, respectively, than male patients in our cohort (AMI: ORf=3.96, 95% CI=3.73-4.2 vs ORm=2.8, 95% CI=2.69-2.91, *P*_SD<.0001; CHD: ORf=4.27, 95% CI=4.11-4.44 vs ORm=3.74, 95% CI=3.67-3.81, *P*_SD<.0001). This effect was especially prominent in the age group of 50-79 years compared to younger patients. There is a tendency that acute kidney disease and CKD are diagnosed after AMI and CHD; see Figure 2.

### Nicotine Dependence

Nicotine abuse had a significantly higher associated AMI and CHD risk for female patients than male patients (AMI: ORf=10.14, 95% CI=9.66-10.64 vs ORm=6.68, 95% CI=6.51-6.84, *P*_SD<.0001; CHD: ORf=6.16, 95% CI=5.96-6.36 vs ORm=4.43, 95% CI=4.35-4.5, *P*_SD<.0001).

### Respiratory Failure

**Figure 3:**
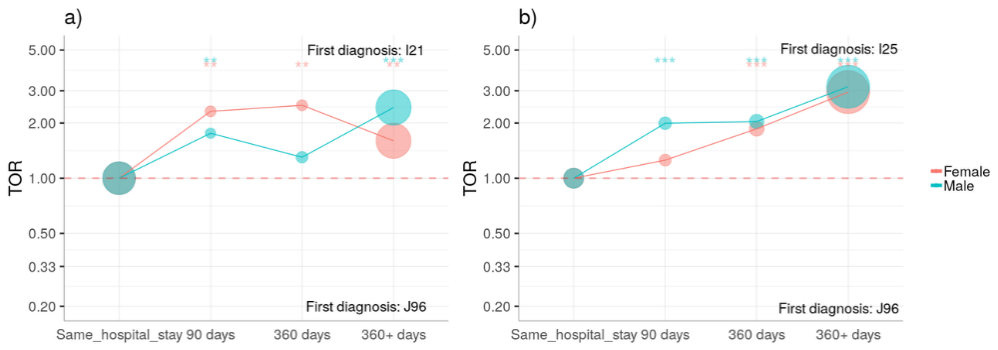
Time directionality analysis for respiratory failure. There is a tendency that patients are first diagnosed with (a) AMI and (b) CHD and then with respiratory failure. Results are shown in Figure 1 for diabetes mellitus. AMI: acute myocardial infarction; CHD: chronic ischemic heart disease; TOR: time order ratio.

Female patients showed a stronger association of respiratory failure with AMI and CHD compared to male patients (AMI: ORf=3.11, 95% CI=2.78-3.48 vs ORm=2.24, 95% CI=2.09-2.4, *P*_SD<.0001; CHD: ORf=2.92, 95% CI=2.75-3.1 vs ORm=2.18, 95% CI=2.1-2.27, *P*_SD<.0001). When splitting the patients in 2 age groups (20-49 years, 50-79 years) to account for potential changes likely related to (peri-)menopause status, respiratory failure was associated with AMI and CHD in the age group of 50-79 years (AMI: ORf=3.11, 95% CI=2.76-3.5 vs ORm=2.23, 95% CI=2.06-2.41, *P*_SD<.0001; CHD: ORf=2.89, 95% CI=2.72-3.07 vs ORm=2.16, 95% CI=2.08-2.25, *P*_SD<.0001) but only with CHD (ORf=6.68, 95% CI=4.34-10.28 vs ORm=2.76, 95% CI=2.27-3.36, *P*_SD<.0001) and not AMI in younger patients. Based on time directionality analysis, we concluded that there is a tendency that respiratory failure is diagnosed after AMI; the same effect can be observed for respiratory failure and CHD (see Figure 3).

### Asthma

Female patients had a significantly higher risk of having asthma and CHD (ORf=2.09, 95% CI=1.96-2.23 vs ORm=1.59, 95% CI=1.5-1.68, *P*_SD<.0001) but not asthma and AMI.

When splitting patients into 2 age groups (20-49 years, 50-79 years) to account for potential differences before and after an age considered likely (peri-)menopausal, asthma and AMI and CHD were significantly more often connected in females in the age group of 50-79 years than in younger patients.

### COPD

COPD and AMI or CHD were more likely to co-occur in female patients than in male patients (AMI: ORf=2.49, 95% CI=2.35-2.63 vs ORm=1.62, 95% CI=1.56-1.68, *P*_SD<.0001; CHD: ORf=2.09, 95% CI=1.96-2.23 vs ORm=1.59, 95% CI=1.5-1.68, *P*_SD<.0001). COPD was more prominently associated with AMI or CHD in the age group of 50-79 years than in younger patients.

### Lipid Metabolism Disorders

Lipid metabolism disorders were associated with an excess risk for AMI and CHD in male than in female patients in our analysis (AMI: ORf=6.3, 95% CI=6.1-6.5 vs ORm=7.21, 95% CI=7.07-7.35, *P*_SD<.0001; CHD: ORf=6.68, 95% CI=6.57-6.79, ORm=8.31, 95% CI=8.21-8.41, *P*_SD<.0001).

## Discussion

### Principal Findings

The results of this analysis demonstrated that except for lipid metabolism disorders, the risk factors overweight and obesity, diabetes mellitus, acute kidney disease and CKD, nicotine dependence, respiratory failure, asthma, and COPD display a stronger connection to CHD and AMI in women than in men.

Obesity predisposes to a multitude of comorbidities, many of which have a negative impact on CVD risk. As women are more likely to be obese [15], these results might be useful to improve screening practices. However, these data contrast a 2015 meta-analysis that concluded that there was no evidence of an SD in CVD risk associated with the body mass index (BMI) [16]. The discrepancy between the meta-analysis and our calculations might stem from coding practices with the ICD-10 code E66 (“overweight and obesity”) preferably being used in patients with a high BMI, whereas the meta-analysis by Mongraw-Chaffin et al related a continuous BMI to CVD risk and, thus, included lower BMI categories as well [16]. An important consideration in overweight and obesity is body fat distribution, often measured in waist-to-hip distribution, as it can predict CVD risk [17].

Before menopause, the more favorable body fat distribution in the lower-body subcutaneous areas might mitigate CVD risk in females. The menopausal loss of ovarian hormones induces a redistribution of body fat to a more visceral, less favorable distribution [18]. In this analysis, the effect of sex on the association between overweight and obesity and AMI and CHD risk was more prominent in the age group of 50 years and above, which potentially corroborates research stating a less favorable distribution after menopause.

Similar to overweight and obesity, females showed a stronger association of diabetes mellitus with AMI or CHD compared to males. The increased risk for female patients with diabetes mellitus to develop AMI or CHD in this study is a well-researched finding, as females with diabetes mellitus lose their “female protection” against CVD [19]. Diabetes mellitus does not only double CVD risk but rather adds an additional 44% risk to females compared to males [6,7].

We found similar increased ORs in female patients with acute kidney disease and CKD who were also more likely to be diagnosed with AMI or CHD than in male patients in our cohort. The complex relationship between CKD and CVD probably results from overlapping risk factors and clustering of unspecific CVD risk factors, such as hypertension, diabetes mellitus, dyslipidemia, and CKD-specific factors (eg, anemia, volume overload) [20]. In general, women have a slightly higher prevalence of CKD, which can most likely be explained by the longer life expectancy of women paired with the age-related decline in kidney function [21]. However, studies show a gender gap in CVD among CKD patients, which emerges in the early nondialysis stages of CKD, with a CVD prevalence of 17.9% in men and 20.4% in women [22]. In studies involving STEMI patients, females had a 5 times greater OR to be diagnosed with renal failure (defined as estimated glomerular filtration rate<60 mL/min), which cannot be fully explained by the marginally higher prevalence of CKD in women [23,24].

Concerning respiratory diseases, females had a 39% or 34% (54% or 31%) increased OR to be diagnosed with AMI or CHD, respectively, than males when they had respiratory failure (COPD). Respiratory failure was significantly more associated with AMI or CHD in women than in men in the age group of 50-79 years compared to younger patients. In younger patients, the effect was only visible in patients with CHD but not AMI. Furthermore, women had a significantly higher risk of having asthma and CHD but not asthma and AMI than men. Accordingly, asthma has been associated with a modest increase in CHD risk in females in a previous study [25]. A possible explanation lies in differences in sex hormones. Estrogen increases and testosterone decreases airway inflammation in asthma. Correspondingly, females begin to display increased asthma symptoms after puberty. The impact of changes in sex hormones levels during menstruation, pregnancy, and menopause are less clear [26]. The higher levels of obesity in females could impact levels of systemic inflammation as well and lead to a relatively increased asthma risk [27]. Moreover, women are more prone to adult asthma and more likely to have severe asthma [26], and an impaired forced expiratory pressure in 1 s (FEV_1_) is associated with a slightly higher hazard ratio (HR) for ischemic heart disease in women than in men [28]. To some extent, we cannot entirely rule out a systematic bias, as smokers with respiratory symptoms tend to be diagnosed with asthma if they are female and with COPD if male. However, even a small increase in the relative incidence of CHD associated with a diagnosis of asthma would have a significant impact due to the commonality of asthma. Further cohort studies are, thus, needed to fully understand the relationship between asthma, FEV_1_, and CHD and be able to take preventative measures [29].

Like in patients with asthma, COPD and AMI or CHD were more likely to co-occur in women than in men. A Finnish national health examination concluded that signs of obstruction in a spirometer at age 30–49 years appears to predict a major coronary event (adjusted HR=4.21) in women only. COPD is the fourth-leading cause of death globally; approximately 50% of those deaths can be attributed to a cardiovascular event (eg, myocardial infarction). With 9.23%, COPD is more prevalent in males than in females (6.95%) [30]; however, the relative prevalence of COPD is rising in women, which is usually explained by the delayed rise of nicotine abuse prevalence among women. Another potential explanation is the susceptibility to COPD risk factors, most importantly nicotine abuse, which appears to be higher in women. Women with COPD are younger and have smoked considerably less than their male counterparts [11]. As nicotine abuse is more detrimental for women in terms of CVD risk as well, it is a crucial shared risk factor for COPD and CVD [30]. Nicotine abuse alone has a significantly higher associated AMI and CHD risk for females than males, which supports the conclusion of a meta-analysis by Huxley and Woodward [3]. Although smoking prevalence is declining worldwide [31], these results are still critical, for instance, considering the high smoking rates among women in high-income countries (16.4%) [31].

Lipid metabolism disorders were the only risk factor with an extensive gender gap in relative AMI and CHD risk associated with an excess risk for males in our analysis. Correspondingly, total cholesterol displayed a higher RR for CVD for men in a meta-analysis as well [32]. At least some of the risk mitigation can be attributed to the less proatherogenic lipid profile of premenopausal women. Specifically, women have relatively more high-density lipoprotein (HDL), less low-density lipoprotein (LDL), but on average larger LDL particles, lower total triglycerides, and circulating very low-density lipoprotein (VLDL) in both smaller concentration and size [33].

### Limitations and Strengths

The analysis is based on a large dataset containing over 45,000,000 hospital diagnoses of the whole Austrian population from 1997 to 2014. The size of the hospital dataset is a clear strength; however, outpatient visits were not recorded. Patients had to have been admitted to a hospital at least once to be included in the analysis. As it is usually the case with medical claims data, our results are likely to be affected by missing diagnoses (in particular, diseases typically not treated in an inpatient setting) and wrong disease classifications. However, nonsystematic errors, for instance, randomly missing diagnoses, do not play a major role, as even if many data points would be missing, the larger the sample size, the more likely one is to still be able to statistically identify an existing correlation. This, of course, does not necessarily apply in the case of systematic errors in the data. Due to the character of this analysis, which is solely based on disease codes, we cannot rule out unobserved confounding factors related to gender aspects. Furthermore, repeated observations of patients over 12 years allowed us to perform a time directionality analysis to identify whether it is more likely that disease A increases the risk for diseases B or whether B is a risk factor for A. However, given the purely observational nature of our dataset, no statements on causality can be made based on this analysis. We chose age 50 as a cutoff for before and after (peri-)menopause as we did not have access to hormone levels or gynecological history; the unreliability of this strict cutoff is a limitation of this analysis.

### Conclusion

Although all the discussed factors increase the risk for CVD in both sexes, nicotine abuse, diabetes mellitus, renal failure, obesity and overweight, and respiratory diseases were relatively more associated with AMI and CHD risk in women in this analysis. Only lipid metabolism disorders displayed the opposite relationship with AMI and CHD. As the inflammatory effect of sex hormones is believed to be a strong influencing factor for SDs in respiratory diseases, we hypothesized that these differences might be age related and change during menopause. Accordingly, SDs in the age group of over 50 years were more prominent than in under 50-year-olds. Further analyses, especially prospective studies, are needed to investigate this topic in detail. However, taken together, these results underline the importance of CVD-screening practices, specifically in women with the above-mentioned risk factors, and emphasize that physicians should be aware of the sex-specific excess risk for AMI and CHD associated with some but not all of their comorbidities.

### Data Availability

The data that support the findings of this study are available from the Austrian Ministry of Health, but restrictions apply to the availability of these data, which were used under license for the current study and so are not publicly available. Data are, however, available from the authors upon reasonable request and with permission of the Austrian Ministry of Health.

## Abbreviations

AMI: acute myocardial infarction
BMI: body mass index
CHD: chronic ischemic heart disease
CI: confidence interval
CKD: chronic kidney disease
COPD: chronic obstructive pulmonary disease
CVD: cardiovascular disease
FEV1: forced expiratory pressure in 1 s
HDL: high-density lipoprotein
HR: hazard ratio
ICD-10: International Classification of Diseases, Tenth edition
LDL: low-density lipoprotein
ORm: odds ratio males
ORf: odds ratio females
P_SD: sex difference P-value
RR: relative risk
SD: sex difference
SE: standard error
STEMI: ST-elevation myocardial infarction
TOR: time order ratio
VLDL: very low-density lipoprotein
WHO: World Health Organization

## References

[1] Merz AA, Cheng S (2016). Sex differences in cardiovascular ageing. Heart.

[2] Wang Y, O'Neil A, Jiao Y, Wang L, Huang J, Lan Y, Zhu Y, Yu C (2019). Sex differences in the association between diabetes and risk of cardiovascular disease, cancer, and all-cause and cause-specific mortality: a systematic review and meta-analysis of 5,162,654 participants. BMC Med.

[3] Huxley R, Woodward M (2011). Cigarette smoking as a risk factor for coronary heart disease in women compared with men: a systematic review and meta-analysis of prospective cohort studies. Lancet.

[4] Peters S, Huxley Rachel R, Woodward Mark (2014). Diabetes as risk factor for incident coronary heart disease in women compared with men: a systematic review and meta-analysis of 64 cohorts including 858,507 individuals and 28,203 coronary events. Diabetologia.

[5] Deischinger Carola, Dervic Elma, Leutner Michael, Kosi-Trebotic Lana, Klimek Peter, Kautzky Alexander, Kautzky-Willer Alexandra (2020). Diabetes mellitus is associated with a higher risk for major depressive disorder in women than in men. BMJ Open Diabetes Res Care.

[6] Woodward M, Peters Sanne A E, Huxley Rachel R (2015). Diabetes and the female disadvantage. Womens Health (Lond).

[7] Kautzky-Willer A, Harreiter Jürgen, Pacini Giovanni (2016). Sex and gender differences in risk, pathophysiology and complications of type 2 diabetes mellitus. Endocr Rev.

[8] Villar E, Remontet L, Labeeuw M, Ecochard R (2007). Effect of age, gender, and diabetes on excess death in end-stage renal failure. JASN.

[9] Gevaert S, De Bacquer Dirk, Evrard Patrick, Renard Marc, Beauloye Christophe, Coussement Patrick, De Raedt Herbert, Sinnaeve Peter R, Claeys Marc J (2013). Renal dysfunction in STEMI-patients undergoing primary angioplasty: higher prevalence but equal prognostic impact in female patients; an observational cohort study from the Belgian STEMI registry. BMC Nephrol.

[10] Cepelis A, Brumpton Ben M, Laugsand Lars E, Dalen Håvard, Langhammer Arnulf, Janszky Imre, Strand Linn B (2019). Asthma, asthma control and risk of acute myocardial infarction: HUNT study. Eur J Epidemiol.

[11] Alonso T, Sobradillo P, de Torres Jp (2017). Chronic obstructive pulmonary disease in women. Is it different?. Archivos de Bronconeumología (English Edition).

[12] Sawalha S, Hedman Linnea, Backman Helena, Stenfors Nikolai, Rönmark Eva, Lundbäck Bo, Lindberg Anne (2019). The impact of comorbidities on mortality among men and women with COPD: report from the OLIN COPD study. Ther Adv Respir Dis.

[13] Deischinger C, Dervic E, Kaleta M, Klimek P, Kautzky-Willer A (2021). Diabetes mellitus is associated with a higher relative risk for Parkinson’s disease in women than in men. JPD.

[14] Kuritz S, Landis J R, Koch G G (1988). A general overview of Mantel-Haenszel methods: applications and recent developments. Annu Rev Public Health.

[15] Garawi F, Devries K, Thorogood N, Uauy R (2014). Global differences between women and men in the prevalence of obesity: is there an association with gender inequality?. Eur J Clin Nutr.

[16] Mongraw-Chaffin M, Peters Sae, Huxley Rr, Woodward M (2015). The sex-specific association between BMI and coronary heart disease: a systematic review and meta-analysis of 95 cohorts with 1·2 million participants. Lancet Diabetes Endocrinol.

[17] Canoy D, Boekholdt Sm, Wareham N, Luben R, Welch A, Bingham S, Buchan I, Day N, Khaw K (2007). Bodyfat distribution and risk of coronary heart disease in men and women in the European prospective investigation into cancer and nutrition in Norfolk cohort. Circulation.

[18] Svendsen O, Hassager C, Christiansen C (1995). Age- and menopause-associated variations in body composition and fat distribution in healthy women as measured by dual-energy x-ray absorptiometry. Metabolism.

[19] Norhammar A, Schenck-Gustafsson K (2013). Type 2 diabetes and cardiovascular disease in women. Diabetologia.

[20] Liu M, Li XC, Lu L, Cao Y, Sun RR, Chen S, Zhang P-y (2014). Cardiovascular disease and its relationship with chronic kidney disease. Eur Rev Med Pharmacol Sci.

[21] Carrero J, Hecking Manfred, Chesnaye Nicholas C, Jager Kitty J (2018). Sex and gender disparities in the epidemiology and outcomes of chronic kidney disease. Nat Rev Nephrol.

[22] KDOQI (2007). KDOQI clinical practice guidelines and clinical practice recommendations for diabetes and chronic kidney disease. Am J Kidney Dis.

[23] Sederholm Lawesson Sofia, Tödt Tim, Alfredsson Joakim, Janzon Magnus, Stenestrand Ulf, Swahn Eva (2011). Gender difference in prevalence and prognostic impact of renal insufficiency in patients with ST-elevation myocardial infarction treated with primary percutaneous coronary intervention. Heart.

[24] Sederholm Lawesson Sofia, Alfredsson Joakim, Szummer Karolina, Fredrikson Mats, Swahn Eva (2015). Prevalence and prognostic impact of chronic kidney disease in STEMI from a gender perspective: data from the SWEDEHEART register, a large Swedish prospective cohort. BMJ Open.

[25] Iribarren C, Tolstykh Irina V, Eisner Mark D (2004). Are patients with asthma at increased risk of coronary heart disease?. Int J Epidemiol.

[26] Fuseini H, Newcomb Dawn C (2017). Mechanisms driving gender differences in asthma. Curr Allergy Asthma Rep.

[27] Zhang P, Zein Joe (2019). Novel insights on sex-related differences in asthma. Curr Allergy Asthma Rep.

[28] Hole D, Watt G C, Davey-Smith G, Hart C L, Gillis C R, Hawthorne V M (1996). Impaired lung function and mortality risk in men and women: findings from the Renfrew and Paisley prospective population study. BMJ.

[29] Hubbard R, West Joe (2004). Commentary: does the presence of asthma increase the incidence of coronary heart disease?. Int J Epidemiol.

[30] Ntritsos G, Franek J, Belbasis L, Christou Ma, Markozannes G, Altman P, Fogel R, Sayre T, Ntzani Ee, Evangelou E (2018). Gender-specific estimates of COPD prevalence: a systematic review and meta-analysis. COPD.

[31] World Health Organization (2019). Report on Global Tobacco Epidemic.

[32] Peters S, Singhateh Yankuba, Mackay Diana, Huxley Rachel R, Woodward Mark (2016). Total cholesterol as a risk factor for coronary heart disease and stroke in women compared with men: a systematic review and meta-analysis. Atherosclerosis.

[33] Wang X, Magkos Faidon, Mittendorfer Bettina (2011). Sex differences in lipid and lipoprotein metabolism: it's not just about sex hormones. J Clin Endocrinol Metab.

